# Reply: “Comment on: Endogenous Ouabain and Related Genes in the Translation from Hypertension to Renal Diseases”

**DOI:** 10.3390/ijms20030542

**Published:** 2019-01-28

**Authors:** Marco Simonini, Paola Casanova, Lorena Citterio, Elisabetta Messaggio, Chiara Lanzani, Paolo Manunta

**Affiliations:** Genomics of Renal Disease and Hypertension Unit, IRCCS San Raffaele Scientific Institute, Università Vita Salute San Raffaele, 20132 Milan, Italy; simonini.marco@hsr.it (M.S.); casanova.paola@hsr.it (P.C.); citterio.lorena@hsr.it (L.C.); messaggio.elisabetta@hsr.it (E.M.); lanzani.chiara@hsr.it (C.L.)

## Introduction

In his recent letter, Dr. Fürstenwerth expressed his perplexity regarding endogenous ouabain (EO) existence and functions [1].

The clinical history of ouabain and related cardiac glycosides has been widely reviewed, starting from Withering in 1785, and progressing through the German literature in the last century. The genomics era has allowed the development of a specific work-stream regarding EO, which is the focus of our review. Indeed, we have presented significant experimental evidence linking specific gene polymorphisms with EO, and their impact on the function of vasculature and other tissues [2].

In our recent work [2], we only described the effect of Endogenous ouabain in different pathophysiological contexts. We avoided describing the potential therapeutic effects of exogenous cardiotonic steroids because they were not the main aim of the paper. Indeed endogenous and exogenous ouabain should be different compounds, with different plasma concentrations (EO usually between pM–nM/L; exogenous ouabain usually μM–mM/L) and very different cellular effects [3,4,5].

EO was first isolated from, and identified in, human plasma over 25 years ago [3]. Despite confirmation in humans and other mammals with mass spectrometry (MS), nuclear magnetic resonance (NMR), and combined liquid chromatography (LC)-immunology methods [4,5], the existence of plasma EO per se has remained controversial [6]. New analytical studies and related findings are very relevant in this regard. For example, the use of high-performance liquid chromatography, coupled with offline multistage MS (MS2, and MS3), to examine the effects of pregnancy and of central angiotensin (Ang) II infusion on EO in rat plasma, led to the detection of EO and two other novel EO isomers [7,8]. These isomers have distinct chromatographic polarity compared to EO, while both have major MS2 and MS3 product ion spectra that are essentially indistinguishable from those of EO. Furthermore, both isomers bind to the anti-Ouabain antibody routinely employed in our radioimmunoassay (RIA), although affinity for the second isomer is at least an order of magnitude weaker that for EO. Both of these new isomers appear to be regulated independently from EO and may vary according to gender, age, and disease. Importantly, neither isomer was previously described nor is detectable in commercial sources of (plant) ouabain. Finally, recent work has confirmed that adrenal gland rat cells were able to produce and secrete EO compound [9].

The existence of EO in human plasma remains controversial, fuelled in part by Baecher et al. [10], who were unable to detect EO in human plasma using LC-MS. It is worth noting that the primary conclusion, as well as other circumstances surrounding the claim of Baecher et al., have been questioned [11,12]. Moreover, the plasma extracts used by Baecher and colleagues tested positive for EO with a well-documented Radiommunoassay (RIA) run in our laboratory [13,14]. These RIA data are significant because, in prior studies, EO has been routinely detected when the same sample extracts were subjected to LC-RIA and LC-MS [15,16]. Furthermore, the critical analysis of the work performed on EO includes evidence from independent laboratories in several continents gathered from 1990 to 2009, which is consistent with an endogenous source of endogenous ouabain [11] in the circulation.

Starting from 2009 [17,18,19], steroid biosynthesis, genetic polymorphisms, and renal function have been linked to EO in a variety of clinical settings, particularly with regard to the previously shown genes involved in EO synthesis: the *Lanosterol Synthase* (LSS) gene polymorphism at the rs2254524 AA vs. CC [20]. LSS rs2254524 AA polymorphism was associated with: (1) an increase in the production of EO after transfection in human adrenal cells; (2) an increase of EO in renal tissue; and (3) a faster decrease of GFR in spite of similar levels of blood pressure [21]. These results are consistent with both (4) an increase in the incidence of Acute kidney Injury (AKI) after cardiac surgery [22] in patients carrying LSS rs2254524 AA polymorphism; and (5) podocyte damages after incubation with ouabain in animal models [23]. The latter evidence is prevented by the selective ouabain inhibitor, Rostafuroxin [24]. Finally, (6) in naïve hypertensive patients Rostafuroxin normalizes Blood Pressure (BP) in LSS AA carriers, but it is inactive in CC carriers [20]. This is consistent with (7) specific data [25] showing the pressor effects of ouabain [26] in rats associated with the peculiar damage [27], and with (8) the presence of cell functional changes that are all prevented by Rostafuroxin [28]. These 8 groups of independent findings gathered from rats and humans, both at the genetic cell and whole-body level, certainly substantiate the above data on EO plasma levels and are also relevant for establishing the scientific truth. Further evidence adding to the relationship between circulating EO and certain genetic polymorphisms (and highlighting this system as a target in the era of precision medicine) is under evolution [21].

In contrast to the in vivo cardio-protective effects of exogenous ouabain in rats, in our peer reviewed clinical studies we repeatedly observed that higher levels of circulating EO are associated with worsening outcomes among patients with cardiac and renal dysfunction. We should agree that association is especially striking, but now it is supported by many clinical data collected during more than twenty years of observation [13,14,25,29,30,31].
